# Oral health and caries/gingivitis-associated factors of adolescents aged 12–15 in Shandong province, China: a cross-sectional Oral Health Survey

**DOI:** 10.1186/s12903-021-01640-x

**Published:** 2021-06-05

**Authors:** Meng Zhang, Jing Lan, Tiantian Zhang, Wenshuang Sun, Panpan Liu, Zhifeng Wang

**Affiliations:** Department of Pediatricss Dentistry, School and Hospital of Stomatology, Cheeloo College of Medicine, Shandong University and Shandong Provincial Key Laboratory of Oral Tissue Regeneration and Shandong Engineering Laboratory for Dental Materials and Oral Tissue Regeneration, No.44-1 Wenhua Road West, Jinan, 250012 Shandong China

**Keywords:** Dental caries, Gingivitis, Adolescents, Oral health, Associated factors

## Abstract

**Background:**

We aimed to analyse the oral health status of adolescents in Shandong province, including dental caries and gingivitis, and their associated factors.

**Methods:**

Adolescents aged 12–15-years in Shandong province were recruited. Caries and gingival status were assessed following the World Health Organisation diagnostic criteria. Information including the sociodemographic, oral hygiene knowledge, attitudes and practices were collected through the questionnaire. Chi-square test and multivariate logistic regression analysis were used to investigate the oral diseases associated factors.

**Results:**

In total, 3868 students (50.2% males) were enrolled. Of these, 39.9% of the participants experienced caries, and 81.7% and 31.3% had calculus and bleeding gingival, respectively. Multivariate logistic regression analysis revealed that there was an association between dental caries and toothaches, dental visits and sleeping troubles caused by oral problems (*P* < 0.024). A low-frequency of brushing, high sugar consumption and no flossing were more associated with calculus formation and gingival bleeding (*P* < 0.008).

**Conclusion:**

Compared to caries, worse gingival condition was more prevalent among adolescents in Shandong province. Brushing behaviour is associated with gingivitis, while dental visits and toothaches are associated with caries. Hence, prevention-oriented dental visits and oral hygiene training are strongly recommended to improve oral health status.

**Supplementary Information:**

The online version contains supplementary material available at 10.1186/s12903-021-01640-x.

## Background

Dental caries and gingivitis are the most prevalent oral diseases and bring a huge economic burden [1, 2]. The adolescents aged 12–15 are in the early stages of permanent dentition. Data shows that 38.5–44.4% of adolescents in China have dental caries [3], while 61% and 67.3% of adolescents have gingival bleeding and calculus respectively [4]. This unfavorable oral condition would make some negative effects on the lifespan of permanent dentition. Hence, it is really important to develop good oral hygiene behaviours and lifestyle habits to improve oral health condition during this period [5]. In other words, understanding and controlling the oral diseases associated factors during this period would help adolescents better maintain their oral health.

Compared to early childhood caries, limited data are available on the assessment of oral health status in the adolescents aged 12–15. Importantly, the oral habits and ideology formed by adolescents are related to their current and future oral health [6, 7]. Poor oral hygiene is closely associated with dental caries and gingival inflammation [8]. At the early stage of permanent dentition, if the associated factors are not evaluated, and the intervention measures are not implemented in time to correct poor habits [9, 10], these irreversible oral problems will affect the life of the teeth and even the person's general quality of life [11–13]. Hence, some studies have started to pay more attention on the association of age, sex, region, socioeconomic conditions, personal and professional dental care, oral hygiene behaviours, and dental anxiety with dental caries, gingivitis and oral health-related quality of life in adolescents [11, 12, 14, 15]. These studies provided evidence and reference for designing oral health prevention strategies [16].

However, no data is available for the adolescents in Shandong province. Shandong, as one of the birthplaces of the ancient Chinese civilisation, has nearly 100 million inhabitants, and they have a special dietary habit (the major staple food is flour). Hence, this study aimed (1) to describe the prevalence of dental caries and gingivitis in 12–15-year-old adolescents in Shandong province and (2) to identify the oral diseases associated factors to provide reference for the improvement of oral health education.

## Methods

This cross-sectional study uses a part of the 4th National Oral Health Survey conducted in China. All parents gave their informed consent for inclusion before their children participated in the study. The study was conducted in accordance with the Declaration of Helsinki, and the protocol was approved by the Ethics Committee of the Chinese Stomatological Association (No. 2014-003).

The sample size was calculated based on the data of the 3rd National Oral Health Survey in 2005, in which the prevalence of dental caries for those aged 12 years was 28.9%. The design effect (deff = 4.5), significance level (α = 5%), margin of error (δ = 10%) and non-response rate (20%) were also included in the following formula:

$$n = deff\frac{{u_{\alpha } /2^{2} }}{{\delta^{2} }}p\left( {1 - p} \right).$$

In total, 28,365 12–15-year-old adolescents should be recruited in 31 provinces across the country [17]. Hence, at least 3660 individuals should be enrolled in this study. A multistage cluster sampling method was adopted to select the sample population. The probability proportion to size sampling determined that two urban and two rural samples should be used. Cluster and quota sampling determined the three primary high schools in each region. Three hundred and twenty students (80 for each age group) from each school were included [18].

After giving their informed consent, the students completed the questionnaire survey and underwent an oral examination. The questionnaire collected information on the students’ personal and family demographics, including age, gender, region (including Huancui, Hedong, Shouguang, Pingyi), father/mother’s education level, whether they were an only child or had siblings, and the following:

1. Oral hygiene knowledge, including the impact of brushing, bacteria, sugar, fluoride, pit and fissure sealing, and other factors that affect the teeth and gingiva.
2. Oral hygiene attitude, mainly to evaluate whether they believe that oral health is important.
3. Oral hygiene behaviours, including brushing habits, frequency of snacking, smoking, dentist visits, and trauma.
4. Troubles caused by oral problems, including eating, talking, brushing, working, schooling, sleeping, smiling, easily troubled, and communicating.

Please refer to the reference [3] for detail information about the questionnaire.

Three questionnaire interviewers who underwent the screening, training and certification by the nation were arranged to collect the above information as efficiently and unbiasedly as possible. The coincidence rate of the questionnaire answers between each interviewers and trainer must exceed 95%. The questionnaires were completed independently by students in the classroom. During this process, one staff was arranged to explain each question publicly to all adolescents in the same classroom to guild them give as the most reliable answers as possible. Parallelly, two other staffs were arranged to clarify personal doubts. At last, each questionnaire would be rechecked by corresponding staff to avoid omissions and vacancies. All procedures were carefully conducted to minimize potential bias.

All clinical examinations were implemented at schools with external equipment (portable dental chair, disposable dental mirror, ball-ended community periodontal index probe, and intraoral light-emitting diode light) [18]. The following indices were applied according to the criteria recommended by the World Health Organisation [19]. The dental caries (DMFT), calculus (CI) and gingival bleeding (GB) indexes assessed decay, missing teeth caused by decay, filled teeth and the gingival health status. All teeth present were gently probed with a CPI probe at six sites, including mesial, mid, and distal on both buccal and lingual surfaces. Dental caries, calculus and gingival bleeding were scored as present (1, DMFT/CI/GB > 1) or absent (0, DMFT/CI/GB = 0) and the number of teeth with DMFT, calculus and gingival bleeding were recorded. All oral examinations were performed by three certified examiners who selected from clinical dentists and trained by the nation, and were recorded by trained dental personnel. The clinical practice training for DMFT would be terminated when the Kappa value was greater than 0.8 to ensure the consistency of the inter- and intra-examiners (0.8–0.83 for three examiners).

Data were entered and statistically analysed using IBM SPSS Statistics version 21.0. The percentage of dental caries (DMFT), calculus and gingival bleeding were calculated and statistical analysis. The indicators were all binary variables, and the other independent variables were also categorical. All the variables were independent of each other. Hence, Chi-square, Fisher’s exact and *z* tests for post hoc comparisons, univariable logistic analysis were conducted to explore the relationship between these oral indicators and the sociodemographic and questionnaire variables. To evaluate the caries and gingivitis associated factors in adolescents, a multivariate logistic regression model (method: backward logistic regression) was used. Those variables with *P* ≤ 0.10 obtained through bivariate analysis were included in the final models. A *P* value of < 0.05 was considered statistically significant.

## Results

A total of 3868 students aged 12–15 years were enrolled. The mean age of the participants was 13.95 ± 1.11 and 50.2% were males. As shown in Table 1, 39.9% of participants experienced dental caries (87.7% DT, 0.8% MT, 21.5% FT), and 81.7% and 31.3% of participants had calculus and bleeding gingival, respectively. The mean (SD) value of DT, FT, DMFT, CI, and GB were 0.68 ± 1.22, 0.18 ± 0.77, 0.86 ± 1.47, 5.61 ± 4.91, and 1.58 ± 3.58, respectively (data not shown).

**Table 1:** Statistical significance by demographic variables for dental caries, calculus and gingival bleeding among 12–15-year-old students (n = 3868). N (%)

Variables	Dental caries			Calculus			Gingival bleeding		
	Absence	Presence	*P* value	Absence	Presence	*P* value	Absence	Presence	*P* value
Total	2326 (60.1%)	1542 (39.9%)		708 (18.3%)	3160 (81.7%)		2656 (68.7%)	1212 (31.3%)	
Age group									
12 years	589 (62.7%)^a^	351 (37.3%)^a^	0.041	225 (23.9%)^a^	715 (76.1%)^a^	< 0.001	666 (70.9%)^a^	274 (29.1%)^a^	0.01
13 years	582 (59.2%)^a,b^	401 (40.8%)^a,b^		175 (17.8%)^b^	808 (82.2%)^b^		666 (67.8%)^a,b^	317 (32.2%)^a,b^	
14 years	597 (61.9%)^a^	368 (38.1%)^a^		178 (18.4%)^b^	787 (81.6%)^b^		687 (71.2%)^a^	278 (28.8%)^a^	
15 years	558 (56.9%)^b^	422 (43.1%)^b^		130 (13.3%)^c^	850 (86.7%)^c^		637 (65.0%)^b^	343 (35.0%)^b^	
Gender									
Male	1300 (66.9%)^a^	642 (33.1%)^a^	< 0.001	281 (14.5%)^a^	1661 (85.5%)^a^	< 0.001	1313 (67.6%)	629 (32.4%)	0.155
Female	1026 (53.3%)^b^	900 (46.7%)^b^		427 (22.2%)^b^	1499 (77.8%)^b^		1343 (69.7%)	583 (30.3%)	
Urban and rural									
Urban	1145 (59.2%)	790 (40.8%)	0.222	380 (19.6%)^a^	1555 (80.4%)^a^	0.032	1270 (65.6%)^a^	665 (34.4%)^a^	< 0.001
Rural	1181 (61.1%)	752 (38.9%)		328 (17.0%)^b^	1605 (83.0%)^b^		1386 (71.7%)^b^	547 (28.3%)^b^	
Region									
Shouguang Weifang	540 (56.4%)^a^	418 (43.6%)^a^	< 0.001	187 (19.5%)^a^	771 (80.5%)^a^	0.001	665 (69.4%)^a^	293 (30.6%)^a^	< 0.001
Huancui, Weihai	550 (58.0%)^a^	399 (42.0%)^a^		168 (17.7%)^a^	781 (82.3%)^a^		588 (62.0%)^b^	361 (38.0%)^b^	
Hedong, Linyi	595 (60.3%)^a,b^	391 (39.7%)^a,b^		212 (21.5%)^b^	774 (78.5%)^b^		682 (69.2%)^a^	304 (30.8%)^a^	
Pingyi, Linyi	641 (65.7%)^b^	334 (34.3%)^b^		141 (14.5%)^c^	834 (85.5%)^c^		721 (73.9%)^c^	254 (26.1%)^c^	
An only child or not?									
Yes	913 (62.7%)^a^	542 (37.3%)^a^	0.01	274 (18.8%)	1181 (81.2%)	0.51	997 (68.5%)	458 (31.5%)	0.881
No	1413 (58.6%)^b^	1000 (41.4%)^b^		434 (18.0%)	1979 (82.0%)		1659 (68.8%)	754 (31.2%)	
Father's education level									
Never go to school	10 (62.5%)	6 (37.5%)	0.286	3 (18.8%)	13 (81.3%)	0.712	9 (56.3%)	7 (43.8%)	0.274
Elementary or junior high school	1476 (61.3%)	932 (38.7%)		425 (17.6%)	1983 (82.4%)		1646 (68.4%)	762 (31.6%)	
High school	318 (56.6%)	244 (43.4%)		112 (19.9%)	450 (80.1%)		374 (66.5%)	188 (33.5%)	
College or university	349 (59.9%)	234 (40.1%)		109 (18.7%)	474 (81.3%)		418 (71.7%)	165 (28.3%)	
No father or do not know	173 (57.9%)	126 (42.1%)		59 (19.7%)	240 (80.3%)		209 (69.9%)	90 (30.1%)	
Mother’s education level									
Never go to school	103 (68.7%)^a^	47 (31.3%)^a^	0.005	29 (19.3%)	121 (80.7%)	0.519	103 (68.7%)	47 (31.3%)	0.255
Elementary or junior high school	1545 (61.2%)^b^	978 (38.8%)^b^		446 (17.7%)	2077 (82.3%)		1728 (68.5%)	795 (31.5%)	
High school	197 (53%)^c^	175 (47%)^c^		74 (19.9%)	298 (80.1%)		247 (66.4%)	125 (33.6%)	
College or university	290 (59.1%)^c^	201 (40.9%)^c^		101 (20.6%)	390 (79.4%)		357 (72.7%)	134 (27.3%)	
No mother or do not know	191 (57.5%)^b,c^	141 (42.5%)^b,c^		58 (17.5%)	274 (82.5%)		221 (66.6%)	111 (33.4%)	

^a,b,c^Differences between the row variables, the same mark represents no difference between the two variables

Table 1 summarises the sociodemographic variables associated with DMFT, CI, and GB. As for dental caries, there was a statistically significative association between caries and age (*P* = 0.041), gender (*P* < 0.001), living region (*P* < 0.001), with or without siblings and mother’s education levels (*P* = 0.01 and 0.005). There was no association with urban/rural region and father's education level. As for dental calculus, age (*P* < 0.001), gender (*P* < 0.001) and living region (*P* = 0.032 and 0.001) were significantly associated with dental calculus, while with and without siblings and parent education level were not associated. As for gingivitis, there was a significative association between bleeding and age (*P* = 0.01), living region (P < 0.001). However, gender, with or without siblings and parents’ education levels were not associated with bleeding.

The independent behavioural variables with significant differences associated with DMFT, CI and GB, are summarised in Additional file 1: Tables S1–3. Dental caries was associated with the following variables: teeth brushing habits and brushing over two times per day (Q4 and Q5; *P* = 0.001 and 0.035), higher frequency of sugar consumption (Q9; *P* < 0.042), poorer self-evaluation of oral condition (Q12; *P* < 0.001), toothaches and dentist visits (Q15-18; *P* < 0.001). Furthermore, those students who did not believe that regular oral examinations are necessary (Q20b; *P* = 0.005) and who thought that caries had a negative impact on eating, schooling, sleeping and easily troubled (Q21; *P* < 0.002) were associated with a higher prevalence of dental caries. Those with a higher frequency of brushing (Q5; *P* < 0.001), higher sugar consumption (Q9a; *P* = 0.03), dental visits (Q16; *P* = 0.003), and believed that regular oral examinations are necessary (Q20b; *P* = 0.016) were associated with a lower prevalence of dental calculus. In addition, a high prevalence of GB was associated with a lower frequency of brushing and flossing (Q5 and Q8; *P* = 0.003 and 0.009), poorer self-evaluation of oral condition (Q12; *P* = 0.048), and the belief that gingival bleeding is normal when brushing (Q19a; *P* < 0.019). The univariable logistic regression analysis showed similar results (see Additional file 1: Tables S4–6).

The multivariate logistic regression analysis revealed that adolescents who were older, female, living in an urban environment, had siblings, a poorer self-evaluation of oral condition, experienced toothaches, visited a dentist, and had sleeping disturbance caused by oral problems had a statistically higher prevalence of DMFT (Table 2). Interestingly, no brushing and sugar consumption variables were included in the final DMFT model. In the CI model (Table 3), there was a higher prevalence of dental calculus in those that were older, male, living in Huancui and Pingyi, and did not visit a dentist. Besides, a low-frequency of brushing (1/day: OR: 1.421, 95% CI 1.158–1.742, *P* = 0.001; < 1/day: OR: 1.691, 95% CI 1.209–2.364, *P* = 0.002) and sugar consumption (OR: 1.75, 95% CI 1.161–2.639, *P* = 0.008) were also found to contribute to a higher prevalence of calculus. The GB model (Table 4) revealed that the students who brushed their teeth less than twice a day (1/day: OR: 1.235, 95% CI 1.028–1.483, *P* = 0.024; < 1/day: OR: 1.791, 95% CI 1.373–2.335, *P* < 0.001) and do not use dental floss (OR: 1.928, 95% CI 1.373–2.706, *P* < 0.001) generally experienced worse gingival bleeding. Moreover, age, living region, and the belief that gingival bleeding when brushing is normal were also associated with GB.

**Table 2:** Multivariable logistic regression model for dental caries (n = 3868; method: backward)

Variables	OR	95% CI	*P* value
Age group			
12 years	1	NA	NA
13 years	1.176	0.971–1.425	0.096
14 years	1.045	0.861–1.268	0.653
15 years	1.322	1.092–1.601	0.004
Region			
Shouguang, Weifang	1.397	1.140–1.711	0.001
Huancui, Weihai	1.435	1.158–1.779	0.001
Hedong, Linyi	1.12	0.923–1.360	0.252
Pingyi, Linyi	1	NA	NA
Gender			
Female	1.69	1.471–1.943	< 0.001
Male	1	NA	NA
An only child or not?			
Yes	1	NA	NA
No	1.253	1.065–1.474	0.007
Q12 self-evaluation of oral health condition			
Great	1	NA	NA
Good	1.415	1.0–2.003	0.05
General	1.518	1.080–2.134	0.016
Poor	2.633	1.82–3.896	< 0.001
Severe	3.346	1.783–6.278	< 0.001
Q15 toothache in the past 12 months			
Usually	2.207	1.391–3.501	0.001
Occasionally	1.482	1.264–1.736	< 0.001
Do not remember	0.995	0.795–1.244	0.963
Never	1	NA	NA
Q16 visited a dentist			
Yes	1.728	1.501–1.990	< 0.001
Never	1	NA	NA
Q21f the impact of oral problems on sleeping			
Serious impact	1.538	1.080–2.190	0.017
General impact	0.998	0.749–1.330	0.991
Minor impact	1.193	0.980–1.452	0.079
Unclear	0.756	0.527–1.084	0.128
No impact	1	NA	NA

*OR* odd rates, *CI* confidence interval, *NA* not applicable

**Table 3:** Multivariate logistic regression model for calculus (method: backward)

Variables	OR	95% CI	*P *value
Age group			
12 years	1	NA	NA
13 years	1.566	1.227–2	< 0.001
14 years	1.366	1.074–1.736	0.011
15 years	2.131	1.65–2.753	< 0.001
Region			
Shouguang, Weifang	1.236	0.972–1.573	0.084
Huancui, Weihai	1.456	1.133–1.871	0.003
Pingyi, Linyi	1.554	1.190–2.028	0.001
Hedong, Linyi	1	NA	NA
Gender			
Male	1.57	1.307–1.886	< 0.001
Female	1	NA	NA
Q5 frequency of brushing (n = 3356)			
≥ 2/day	1	NA	NA
1/day	1.421	1.158–1.742	0.001
< 1/day	1.691	1.209–2.364	0.002
Q9a frequency of having sweets			
Seldom or never	1.75	1.161–2.639	0.008
1–3/month	1.37	0.944–1.988	0.098
1/week	1.318	0.951–1.827	0.097
2–6/week	1.311	0.981–1.752	0.067
1/day	1.1	0.8–1.512	0.557
≥ 2/day	1	NA	NA
Q16 whether visited a dentist			
Yes	1	NA	NA
Never	1.249	1.037–1.504	0.019

*OR* odd rates, *CI* confidence interval, *NA* not applicable

**Table 4:** Multivariate logistic regression model for gingival bleeding (method: backward)

Variables	OR	95% CI	*P* value
Age group			
12 years	1.09	0.876–1.356	0.441
13 years	1.27	1.027–1.571	0.028
14 years	1	NA	NA
15 years	1.354	1.1–1.667	0.004
Region			
Shouguang, Weifang	1.288	1.032–1.608	0.025
Huancui, Weihai	2.109	1.686–2.636	< 0.001
Hedong, Linyi	1.233	0.989–1.537	0.063
Pingyi, Linyi	1	NA	NA
Q5 frequency of brushing (n = 3356)			
≥ 2/day	1	NA	NA
1/day	1.235	1.028–1.483	0.024
< 1/day	1.791	1.373–2.335	< 0.001
Q8 frequency of using dental floss			
Never	1.928	1.373–2.706	< 0.001
Occasionally	1	NA	NA
Weekly use	0.642	0.135–3.061	0.579
Daily use	1.24	0.311–4.954	0.76
Q19a is gingival bleeding normal when brushing your teeth?			
Yes	1.516	1.225–1.875	< 0.001
Do not know	1.322	1.025–1.704	0.031
No	1	NA	NA

*OR* odd rates, *CI* confidence interval, *NA* not applicable

## Discussion

This study evaluated the sociodemographic, clinical, oral health knowledge, attitudes, and behavioural factors among 12–15-year-old students in Shandong province and analysed the association between non-clinical variables and the DMFT, CI, and GB indices (clinical variables). The results show that the prevalence of caries in Shandong province is similar to the national level (31.9% vs 41.9%) [3], the detection rate of calculus is higher than it is nationally (81.7% vs 67.3%), while the gingival bleeding rate is lower than nationally (31.3% vs 61.0%) [4]. These inconsistencies may be related to the geographic location of Shandong province, the diet habits and oral health behaviours of adolescents, etc. Therefore, further oral diseases associated factors were explored and analysed in this study.

Dental caries are an age-related disease [20–22], which is relate to the continuous development of oral diseases over time without any intervention [23]. In addition, there is an association between gender and caries. Here, a higher frequency of caries is detected in females, which may be related to their preference for dessert (Q9a, over 2 times/day: male: 43.9%, female: 56.1%, *P* < 0.0001. data not shown), the hormonal fluctuations in puberty and menstruation [24], or even the sex-based diverse plaque microbiome [25]. However, some studies found that males usually have a higher frequency of caries [26, 27]. In other words, the association between gender and caries is been confirmed, but not fixed. Furthermore, gender is also associated with the gingival health. Here, boys tend to have more severe gingival health status than girls, as supported by many studies [28–30]. Boy’s poor oral hygiene habits (Q4, toothbrushing: yes: male vs female: 45.5% vs 54.5%; occasionally or never: male vs female: 80.9% vs 19.1%. *P* < 0.0001; Q5, frequency of brushing: twice daily: male vs female: 38.5% vs 61.5%, *P* < 0.0001. data not shown) may be contribute to this result [6].

In addition, region is also an associated factor related to caries, calculus and gingival bleeding. Especially adolescents living in Weifang and Weihai are more likely to experience dental caries and gingival bleeding than those living in Linyi. This may be results from the special dietary habits in Linyi area. Residents living in Linyi take a hard and tough pancake as their staple food, which is made from coarse grains. The low adhesion and easy friction properties of this food may hinder the accumulation of dental plaque, thereby reducing the prevalence of dental caries in this area.

Unfortunately, these associated factors, including age, gender, and region are all uncontrollable factors, even if there are significant differences. Therefore, stratification is important for the analysis and assessment of the risk factors at different levels. Moreover, an analysis of controllable oral diseases associated variables is more important to provide robust guidance for oral health education content.

The contribution of toothbrushing to remove dental plaque and maintain oral health has been proven [31–33]. However, many of these cross-sectional studies and even longitudinal studies [15, 34, 35] are consistent with our results, and found that there is no association between toothbrushing and dental caries. We speculate that this result may be related to individual’s brushing efficiency, that is, whether the toothbrushing practices get the maximum effect to remove dental plaque [10]. This also explains why girls suffer from dental caries more frequently in this study. Girls prefer desserts, even though they brush their teeth twice a day, they still have a high incidence of dental caries. After all, toothbrushing is already a very common oral health care behaviour in China, but how to ensure and improve the efficiency of individuals toothbrushing is the direction we should strive for now. Currently, an increasing number of prospective intervention studies have confirmed that additional brushing guidance or even just a simple application of plaque disclosing tables can improve oral hygiene [36, 37]. Hence, an assessment of brushing efficiency should be incorporated into future research to estimate the correlation between toothbrushing and dental caries more accurately. In addition, the positive effect of brushing more than twice a day on preventing calculus formation and gingival bleeding were confirmed in our study. Evidence shows that, without considering the efficacy of tooth brushing, correct oral hygiene can help to maintain gingival health [6, 38].

Preventative dental visits are highly recommended [39]. However, in China, there seems to be still a long way to go, as dental visits were generally associated with worse caries status [40, 41], and dental visits for treatment is a vital attribution factor. Better calculus status was detected among adolescents who had visited the dentist (Additional file 1: Table S2, Table 3), which may be related to the time-efficiency of calculus removal. Furthermore, those individuals that had experienced a toothache in the past 12 months, and sleeping troubles caused by oral problems were found to have a more severe caries status (Additional file 1: Table S1, Table 2), so they evaluated themselves as having poor oral health. However, 81.7% of individuals with calculus and 31.3% with gingival bleeding, did not realize that their oral health was very poor. Furthermore, 14.1% of adolescents thought that gingival bleeding was normal when brushing teeth, indicating that compared to caries, some characteristics of gingivitis, especially the formation of calculus, are not often taken seriously or even ignored when adolescents self-evaluate oral health status. If adolescents continue to pay little attention to initial periodontal inflammation, more serious periodontal issues will inevitably appear in the future [8]. Therefore, these aspects should be focussed on when conducting oral health education in the future.

The sufficient sample size and the comprehensive collection of various potential associated factor information enabled this study to reliably evaluate the oral health status of adolescents in Shandong province. However, the present study also had some limitations. First, cross-sectional studies are unable to prove causal inference. Second, the correlation between toothbrushing and caries could not be accurately evaluated because toothbrushing efficiency was not assessed. Third, even though the region that the adolescents lived in was an associated factor that correlated with DMFT, CI, and GB, no information on the familial socioeconomic status was recorded. It is known that familial socioeconomic status is closely linked with oral health status [42–44]. Due to living standard improvements, a simple urban–rural classification does not truly reflect the correlation between socioeconomic status and oral health. Finally, the odds ratios may overestimate the prevalence ratios especially when prevalence is high and thus caution should be taken in interpreting the results.

## Conclusion

In conclusion, compared to caries, worse gingival condition is more prevalent among adolescents in Shandong and its signs are not taken seriously. Age, gender, region, toothaches, dental visits and sleeping troubles caused by oral problems were found to be associated with dental caries in adolescents. A low-frequency of brushing and sugar consumption were associated with the formation of calculus. A low-frequency of brushing and no flossing were associated with gingival bleeding. Preventative dental visits and oral hygiene training are strongly recommended to improve oral health status.

## Supplementary Information

**Additional file 1: Table S1**. Bivariate analysis of potential variables related to the prevalence of dental caries. N (%). **Table S2**. Bivariate analysis of potential variables related to the prevalence of calculus. N (%). **Table S3**. Bivariate analysis of potential variables related to the prevalence of gingival bleeding. N (%). **Table S4**. Binary logistic regression analysis for dental caries (unadjusted). **Table S5**. Binary logistic regression analysis for calculus (unadjusted). **Table S6**. Binary logistic regression analysis for gingival bleeding (unadjusted)

## Data Availability

The datasets used and analyzed during the current study are available from the corresponding author on reasonable request.

## Abbreviations

DMFT: Decayed, missing and filled permanent teeth
CI: Calculus index
GB: Gingival bleeding index
OR: Odd rates
CI: Confidence interval
