# Supplementary material for: Oral health and caries/gingivitis-associated factors of adolescents aged 12–15 in Shandong province, China: a cross-sectional Oral Health Survey
Source: BMC Oral Health. 2021 Jun 5;21:288. doi: 10.1186/s12903-021-01640-x (PMC8178891; doi:10.1186/s12903-021-01640-x)
Supplement: Supplementary file 1 — Additional file 1: Table S1. Bivariate analysis of potential variables related to the prevalence of dental caries. N (%). Table S2. Bivariate analysis of potential variables related to the prevalence of calculus. N (%). Table S3. Bivariate analysis of potential variables related to the prevalence of gingival bleeding. N (%). Table S4. Binary logistic regression analysis for dental caries (unadjusted). Table S5. Binary logistic regression analysis for calculus (unadjusted). Table S6. Binary logistic regression analysis for gingival bleeding (unadjusted) [file 12903_2021_1640_MOESM1_ESM.docx]

**Oral health and risk indicators of early adolescents in Shandong province, China: a cross-sectional Oral Health Survey**

Meng Zhang ^1^, Jing Lan ^1^, Tiantian Zhang ^1^, Wenshuang Sun ^1^, Panpan Liu ^1^, Zhifeng Wang ^1*^.

Table S1: Bivariate analysis of potential variables related to the prevalence of dental caries. N (%)

| Variables | Dental caries | | |
| --- | --- | --- | --- |
|  | Absence | Presence | *P-*value |
| Q4 whether to brush teeth (n=3868) | | | |
| Yes | 1983(59.1%) a | 1373(40.9%) a | 0.001 |
| Occasionally or never | 343(67.0%) b | 169(33.0%) b |  |
| Q5 frequency of brushing (n=3356) | | | |
| ≥2/day | 479(55.6%) b | 383(44.4%) b | 0.035 |
| 1/day | 1266(59.9%) a | 846(40.1%) a |  |
| <1/day | 238(62.3%) a | 144(37.7%) a |  |
| Q9a frequency of having sweet | | | |
| Seldom or never | 232(65.7%) a | 121(34.3%) a | <0.001 |
| 1-3/month | 270(65.1%) a | 145(34.9%) a |  |
| 1/week | 414(62.6%) a | 247(37.4%) a |  |
| 2-6/week | 816(60.6%) a | 531(39.4%) a |  |
| 1/day | 362(54.8%) b | 299(45.2%) b |  |
| ≥2/day | 232(53.8%) b | 199(46.2%) b |  |
| Q9b frequency of having sweet drinks | | | |
| Seldom or never | 573(59.9%) a | 383(40.1%) a | 0.042 |
| 1-3/month | 560(58.1%) a | 404(41.9%) a |  |
| 1/week | 447(58.5%) a | 317(41.5%) a |  |
| 2-6/week | 488(61.6%) a | 304(38.4%) a |  |
| 1/day | 179(68.8%) b | 81(31.2%) b |  |
| ≥2/day | 79(59.8%) a, b | 53(40.2%) a, b |  |
| Q12 self-evaluation of oral condition | | | |
| great | 142(72.4%) a | 54(27.6%) a | <0.001 |
| good | 727(63.6%) b | 416(36.4%) b |  |
| general | 1223(60.8%) b | 787(39.2%) b |  |
| poor | 209(45.7%) c | 248(54.3%) c |  |
| severe | 25(40.3%) c | 37(59.7%) c |  |
| Q15 toothache in the past 12 months | | | |
| Usually | 35(35.4%) a | 64(64.6%) a | <0.001 |
| Occasionally | 1083(54.5%) b | 903(45.4%) b |  |
| Never | 847(68.3%) c | 394(31.7%) c |  |
| do not remember | 361(66.6%) c | 181(33.4%) c |  |
| Q16 visited a dentist | | | |
| Yes | 1051(52.6%) a | 946(47.4%) a | <0.001 |
| Never | 1275(68.1%) b | 596(31.9%) b |  |
| Q17 the last time you visited dentist | | | |
| Within 6 months | 170(42.7%) a | 228(57.3%) a | <0.001 |
| 6-12 months | 219(45.9%) a | 258(54.1%) a |  |
| Over 12 months | 662(59.0%) b | 460(41.0%) b |  |
| Q18 the reason for your last dental visit | | | |
| Consultation | 124(55.1%) a | 101(44.9%) a | <0.001 |
| Prevention | 48(47.5%) b | 53(52.5%) b |  |
| Treatment | 175(36.3%) c | 307(63.7%) c |  |
| Do not know | 42(62.7%) a | 25(37.3%) a |  |
| Q20b do you think regular oral examination is necessary? | | | |
| Yes | 1614(58.6%) b | 1142(41.4%) b | 0.005 |
| No | 36(53.7%) a | 31(46.3%) a |  |
| Doesn't matter | 454(64.5%) b | 250(35.5%) b |  |
| Do not know | 222(65.1%) b | 119(34.9%) b |  |
| Q21a the impact of oral problems on eating | | | |
| Serious impact | 184(52.4%) a | 167(47.6%) a | <0.001 |
| General impact | 364(55.2%) a | 295(44.8%) a |  |
| Minor impact | 656(57.0%) a | 494(43.0%) a |  |
| No impact | 1039(65.2%) b | 554(34.8%) b |  |
| Unclear | 83(72.2%) b | 32(27.8%) b |  |
| Q21e the impact of oral problems on schooling | | | |
| Serious impact | 67(62.0%) a | 41(38.0%) a | 0.002 |
| General impact | 88(50.0%) b | 88(50.0%) b |  |
| Minor impact | 244(55.1%) b | 199(44.9%) b |  |
| No impact | 1839(61.0%) a | 1174(39.0%) a |  |
| Unclear | 88(68.8%) a | 40(31.3%) a |  |
| Q21f the impact of oral problems on sleeping | | | |
| Serious impact | 68(45.6%) a | 81(54.4%) a | <0.001 |
| General impact | 129(56.1%) b | 101(43.9%) b |  |
| Minor impact | 292(54.9%) b | 240(45.1%) b |  |
| No impact | 1729(61.8%) c | 1071(38.3%) c |  |
| Unclear | 108(68.8%) c | 49(31.2%) c |  |
| Q21h the impact of oral problems on easy trouble | | | |
| Serious impact | 105(51.2%) a | 100(48.8%) a | <0.001 |
| General impact | 218(55.6%) a | 174(44.4%) a |  |
| Minor impact | 454(57.3%) a | 338(42.7%) a |  |
| No impact | 1398(61.6%) b | 870(38.4%) b |  |
| Unclear | 151(71.6%) c | 60(28.4%) c |  |

a, b, c: Differences between the row variables, the same mark represents no difference between the two variables.

Table S2: Bivariate analysis of potential variables related to the prevalence of calculus. N (%)

| Variables | Calculus | | |
| --- | --- | --- | --- |
|  | Absence | Presence | *P-*value |
| Q5 frequency of brushing | | | |
| ≥2/day | 209(24.2%) a | 653(75.8%) a | <0.001 |
| 1/day | 363(17.2%) b | 1749(82.8%) b |  |
| <1/day | 58(15.2%) b | 324(84.8%) b |  |
| Q9a frequency of having sweet | | | |
| Seldom or never | 49(13.9%) a | 304(86.1%) a | 0.03 |
| 1-3/month | 72(17.3%) a, b | 343(82.7%) a, b |  |
| 1/week | 119(18.0%) a, b | 542(82.0%) a, b |  |
| 2-6/week | 235(17.4%) a, b | 1112(82.6%) a, b |  |
| 1/day | 139(21.0%) b | 522(79.0%) b |  |
| ≥2/day | 94(21.8%) b | 337(78.2%) b |  |
| Q16 visited a dentist | | | |
| Yes | 401(20.1%) a | 1596(79.9%) a | 0.003 |
| Never | 307(16.4%) b | 1564(83.6%) b |  |
| Q20b do you think regular oral examination is necessary? | | | |
| Yes | 539(19.6%) a | 2217(80.4%) a | 0.016 |
| No | 11(16.4%) a, b | 56(83.6%) a, b |  |
| Doesn't matter | 104(14.8%) b | 600(85.2%) b |  |
| Do not know | 54(15.8%) a, b | 287(84.2%) a, b |  |

a, b: Differences between the row variables, the same mark represents no difference between the two variables.

Table S3: Bivariate analysis of potential variables related to the prevalence of gingival bleeding. N (%)

| Variables | Gingival bleeding | | |
| --- | --- | --- | --- |
|  | Absence | Presence | *P-*value |
| Q5 frequency of brushing | | | |
| ≥2/day | 619(71.8%) a | 243(28.2%) a | 0.003 |
| 1/day | 1462(69.2%) a | 650(30.8%) a |  |
| <1/day | 237(62.0%) b | 145(38.0%) b |  |
| Q8 frequency of dental floss | | | |
| Never | 2453((68.0%) a | 1154(32.0%) a | 0.009 |
| Occasionally | 184(78.0%) b | 52(22.0%) b |  |
| Weekly use | 10(76.9%) a, b | 3(23.1%) a, b |  |
| Daily use | 9(75.0%) a, b | 3(25.0%) a, b |  |
| Q12 self-evaluation of oral condition | | | |
| great | 143(73.0%) a | 53(27.0%) a | 0.048 |
| good | 811(71.0%) a | 332(29.0%) a |  |
| general | 1368(68.1%) a, b | 642(31.9%) a, b |  |
| poor | 296(64.8%) a | 161(35.2%) b |  |
| severe | 38(61.3%) a, b | 24(38.7%) a, b |  |
| Q19a is gingival bleeding normal when brushing your teeth? | | | |
| Yes | 347(63.8%) a | 197(36.2%) a | 0.001 |
| No | 2070(70.2%) b | 880(29.8%) b |  |
| Do not know | 239(63.9%) a | 135(36.1%) a |  |
| Q19g sealing can protect teeth, right? | | | |
| Yes | 315(73.6%) a | 113(26.4%) a | 0.019 |
| No | 233(71.9%) a | 91(28.1%) a |  |
| Do not know | 2108(67.7%) b | 1008(32.3%) b |  |

a, b: Differences between the row variables, the same mark represents no difference between the two variables.

Table S4. Binary logistic regression analysis for dental caries (unadjusted)

| Variables | OR | 95% CI | *P-*value |
| --- | --- | --- | --- |
| Age group | | | |
| 12y | 1 | NA | NA |
| 13y | 1.156 | 0.962-1.389 | 0.121 |
| 14y | 1.034 | 0.859-1.245 | 0.721 |
| 15y | 1.269 | 1.057-1.524 | 0.011 |
|  |  |  |  |
| Region | | | |
| Shouguang, Weifang | 1.486 | 1.236-1.785 | <0.001 |
| Huancui, Weihai | 1.392 | 1.157-1.675 | <0.001 |
| Hedong, Linyi | 1.261 | 1.049-1.516 | 0.013 |
| Pingyi, Linyi | 1 | NA | NA |
|  |  |  |  |
| Gender | | | |
| Female | 1.776 | 1.559-2.023 | <0.001 |
| Male | 1 | NA | NA |
|  |  |  |  |
| An only child or not? | | | |
| Yes | 1 | NA | NA |
| M=No | 1.192 | 1.043-1.363 | 0.010 |
| Mother's education level | | | |
| Never go to school | 1 | NA | NA |
| Elementary or junior high school | 1.387 | 0.973-1.977 | 0.070 |
| High school | 1.974 | 1.304-2.906 | 0.001 |
| College or university | 1.519 | 1.029-2.241 | 0.035 |
| No mother or do not know | 1.618 | 1.076-2.433 | 0.0221 |
| Q4 whether to brush teeth (n=3868) | | | |
| Yes | 1.405 | 1.154-1.711 | 0.001 |
| Occasionally or never | 1 | NA | NA |
| Q5 frequency of brushing (n=3356) | | | |
| ≥2/day | 1.322 | 1.033-1.691 | 0.027 |
| 1/day | 1.104 | 0.882-1.382 | 0.386 |
| <1/day | 1 | NA | NA |
| Q9a frequency of having sweet | | | |
| Seldom or never | 1 | NA | NA |
| 1-3/month | 1.030 | 0.764-1.388 | 0.848 |
| 1/week | 1.144 | 0.873-1.499 | 0.330 |
| 2-6/week | 1.248 | 0.976-1.595 | 0.077 |
| 1/day | 1.584 | 1.211-2.070 | 0.001 |
| ≥2/day | 1.645 | 1.230-2.198 | 0.001 |
| Q9b frequency of having sweet drinks | | | |
| Seldom or never | 1 | NA | NA |
| 1-3/month | 1.079 | 0.900-1.295 | 1.079 |
| 1/week | 1.061 | 0.874-1.288 | 1.061 |
| 2-6/week | .932 | 0.768-1.130 | 0.932 |
| 1/day | .677 | 0.505-0.907 | 0.677 |
| ≥2/day | 1.004 | 0.692-1.455 | 1.004 |
| Q12 self-evaluation of oral health condition | | | |
| great | 1 | NA | NA |
| good | 1.505 | 1.076-2.105 | 0.017 |
| general | 1.692 | 1.222-2.344 | 0.002 |
| poor | 3.120 | 2.170-4.488 | <0.001 |
| severe | 3.892 | 2.144-7.066 | <0.001 |
|  |  |  |  |
| Q15 toothache in the past 12 months | | | |
| Usually | 3.931 | 2.560-6.037 | <0.001 |
| Occasionally | 1.792 | 1.545-2.080 | <0.001 |
| Do not remember | 1.078 | 0.869-1.336 | 0.494 |
| Never | 1 | NA | NA |
| Q16 visited a dentist | | | |
| Yes | 1.926 | 1.689-2.195 | <0.001 |
| Never | 1 | NA | NA |
| Q17 the last time you visited dentist | | | |
| Within 6 months | 1.930 | 1.531-2.433 | <0.001 |
| 6-12 months | 1.695 | 1.366-2.104 |  |
| Over 12 months | 1 | NA | NA |
| Q18 the reason for your last dental visit | | | |
| Consultation |  |  |  |
| Prevention |  |  |  |
| Treatment |  |  |  |
| Do not know | 1 | NA | NA |
| Q20b do you think regular oral examination is necessary? | | | |
| Yes | 1.368 | 0.781-2.397 | 0.273 |
| No | 1.855 | 0.987-3.485 | 0.055 |
| Doesn't matter | 2.947 | 1.737-5.001 | <0.001 |
| Do not know | 1 | NA | NA |
| Q21a the impact of oral problems on eating | | | |
| Serious impact | 2.354 | 1.488-3.723 | <0.001 |
| General impact | 2.102 | 1.360-3.250 | 0.001 |
| Minor impact | 1.953 | 1.278-2.985 | 0.002 |
| No impact | 1.383 | 0.908-2.106 | 0.131 |
| Unclear | 1 | NA | NA |
| Q21e the impact of oral problems on schooling | | | |
| Serious impact | 1.346 | 0.785-2.308 | .280 |
| General impact | 2.200 | 1.366-3.543 | .001 |
| Minor impact | 1.794 | 1.181-2.725 | .006 |
| No impact | 1.404 | 0.960-2.055 | .080 |
| Unclear | 1 | NA | NA |
| Q21f the impact of oral problems on sleeping | | | |
| Serious impact | 1.923 | 1.381-2.678 | <0.001 |
| General impact | 1.264 | 0.964-1.658 | 0.091 |
| Minor impact | 1.327 | 1.101-1.600 | 0.003 |
| Unclear | 0.732 | 0.518-1.035 | 0.078 |
| No impact | 1 | NA | NA |
| Q21h the impact of oral problems on easy trouble | | | |
| Serious impact | 2.397 | 1.598-3.596 | <0.001 |
| General impact | 2.009 | 1.402-2.877 | <0.001 |
| Minor impact | 1.874 | 1.346-2.608 | <0.001 |
| No impact | 1.566 | 1.148-2.137 | 0.005 |
| Unclear | 1 | NA | NA |

OR: odd rates; CI: confidence interval; NA: not applicable

Table S5. Binary logistic regression analysis for calculus (unadjusted)

| variables | OR | 95% CI | *P-*value |
| --- | --- | --- | --- |
| Age group | | | |
| 12y | 1 | NA | NA |
| 13y | 1.453 | 1.164-1.814 | 0.001 |
| 14y | 1.391 | 1.115-1.736 | 0.003 |
| 15y | 2.058 | 1.622-2.610 | <0.001 |
|  |  |  |  |
| Region | | | |
| Shouguang, Weifang | 1.129 | 0.906-1.408 | 0.280 |
| Huancui, Weihai | 1.273 | 1.016-1.595 | 0.036 |
| Pingyi, Linyi | 1.620 | 1.282-2.048 | <0.001 |
| Hedong, Linyi | 1 | NA | NA |
|  |  |  |  |
| Gender | | | |
| Male | 1.684 | 1.426-1.988 | <0.001 |
| Female | 1 | NA | NA |
|  |  |  |  |
| Q5 frequency of brushing (n=3356) | | | |
| ≥2/day | 1 | NA | NA |
| 1/day | 1.542 | 1.272-1.869 | <0.001 |
| <1/day | 1.788 | 1.298-2.462 | <0.001 |
|  |  |  |  |
| Q9a frequency of having sweets | | | |
| Seldom or never | 1.731 | 1.185-2.527 | 0.005 |
| 1-3/month | 1.329 | 0.944-1.870 | 0.103 |
| 1/week | 1.270 | 0.939-1.720 | 0.121 |
| 2-6/week | 1.320 | 1.009-1.726 | 0.043 |
| 1/day | 1.047 | 0.780-1.407 | 0.758 |
| ≥2/day | 1 | NA | NA |
|  |  |  |  |
| Q16 whether visited a dentist | | | |
| Yes | 1 | NA | NA |
| Never | 1.280 | 1.086-1.508 | 0.003 |

OR: odd rates; CI: confidence interval; NA: not applicable

Table S6. Binary logistic regression analysis for gingival bleeding (unadjusted)

| variables | OR | 95% CI | *P-*value |
| --- | --- | --- | --- |
| Age group | | | |
| 12y | 1 | NA | NA |
| 13y | 1.157 | 0.953-1.405 | 0.141 |
| 14y | 0.984 | 0.807-1.199 | 0.870 |
| 15y | 1.309 | 1.080-1.586 | 0.006 |
|  | | | |
| Region | | | |
| Shouguang, Weifang | 1.251 | 1.026-1.525 | 0.027 |
| Huancui, Weihai | 1.743 | 1.435-2.116 | 0.000 |
| Hedong, Linyi | 1.265 | 1.039-1.540 | 0.019 |
| Pingyi, Linyi | 1 | NA | NA |
|  | | | |
| Q5 frequency of brushing (n=3356) | | | |
| ≥2/day | 1 | NA | NA |
| 1/day | 1.133 | 0.951-1.349 | 0.163 |
| <1/day | 1.558 | 1.208-2.010 | 0.001 |
|  | | | |
| Q8 frequency of using dental floss | | | |
| Never | 1 | NA | NA |
| Occasionally | 0.601 | 0.438-0.824 | 0.002 |
| Weekly use | 0.638 | 0.175-2.321 | 0.495 |
| Daily use | 0.709 | 0.191-2.622 | 0.606 |
| Q12 self-evaluation of oral condition | | | |
| great | 1 | NA | NA |
| good | 1.105 | 0.786-1.552 | 0.567 |
| general | 1.266 | 0.911-1.759 | 0.159 |
| poor | 1.468 | 1.015-2.123 | 0.042 |
| severe | 1.704 | 0.935-3.106 | 0.082 |
| Q19a is gingival bleeding normal when brushing your teeth? | | | |
| Yes | 1.335 | 1.102-1.618 | 0.003 |
| Do not know | 1.329 | 1.061-1664 | 0.013 |
| No | 1 | NA | NA |
| Q19g sealing can protect teeth, right? | | | |
| Yes | 1 | NA | NA |
| No | 1.333 | 1.062-1.674 | .013 |
| Do not know | 1.089 | 0.788-1.505 | .607 |

OR: odd rates; CI: confidence interval; NA: not applicable
